# Supplementary material for: Endometrium as Control of Endometriosis in Experimental Research: Assessment of Sample Suitability
Source: Diagnostics (Basel). 2022 Apr 12;12(4):970. doi: 10.3390/diagnostics12040970 (PMC9032605; doi:10.3390/diagnostics12040970)
Supplement: Supplementary file 1 [file diagnostics-12-00970-s001.zip › diagnostics-1591380-supplementary.pdf]

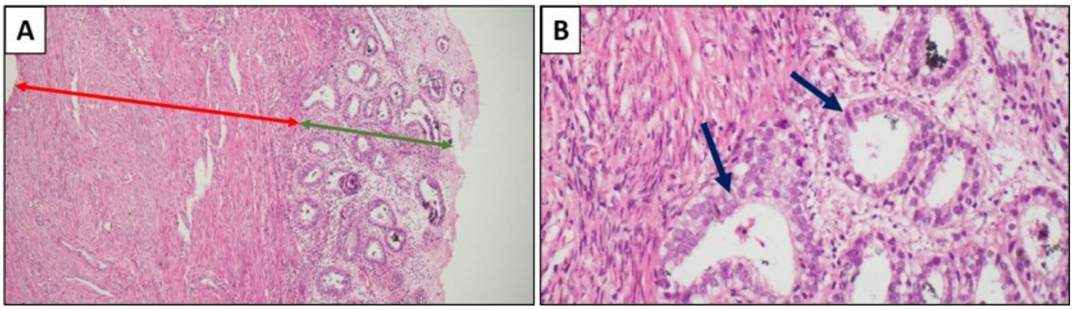


**Supplementary Figure S1.** *Case 1 endometrium – H&E staining, 10× (A) and 40× (B) magnifications of 'Fresh' sample*

*A: Beneath the secretory phase endometrium (green band) sample is composed of a relatively thick myometrium layer (red band)*

*B: Blue arrows show secretory phase endometrial glands.*

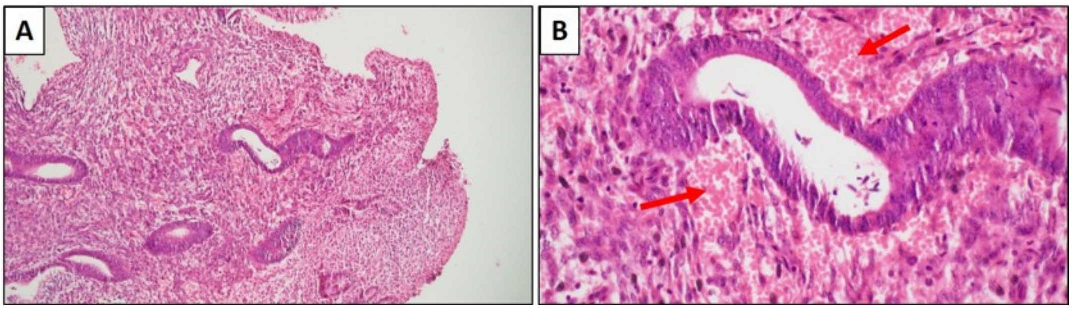


**Supplementary Figure S2.** *Case 2 endometrium – H&E staining, 10× (A) and 40× (B) magnifications of 'Fresh' sample*

*Red arrows show interstitial haemorrhage (artificial injury)*

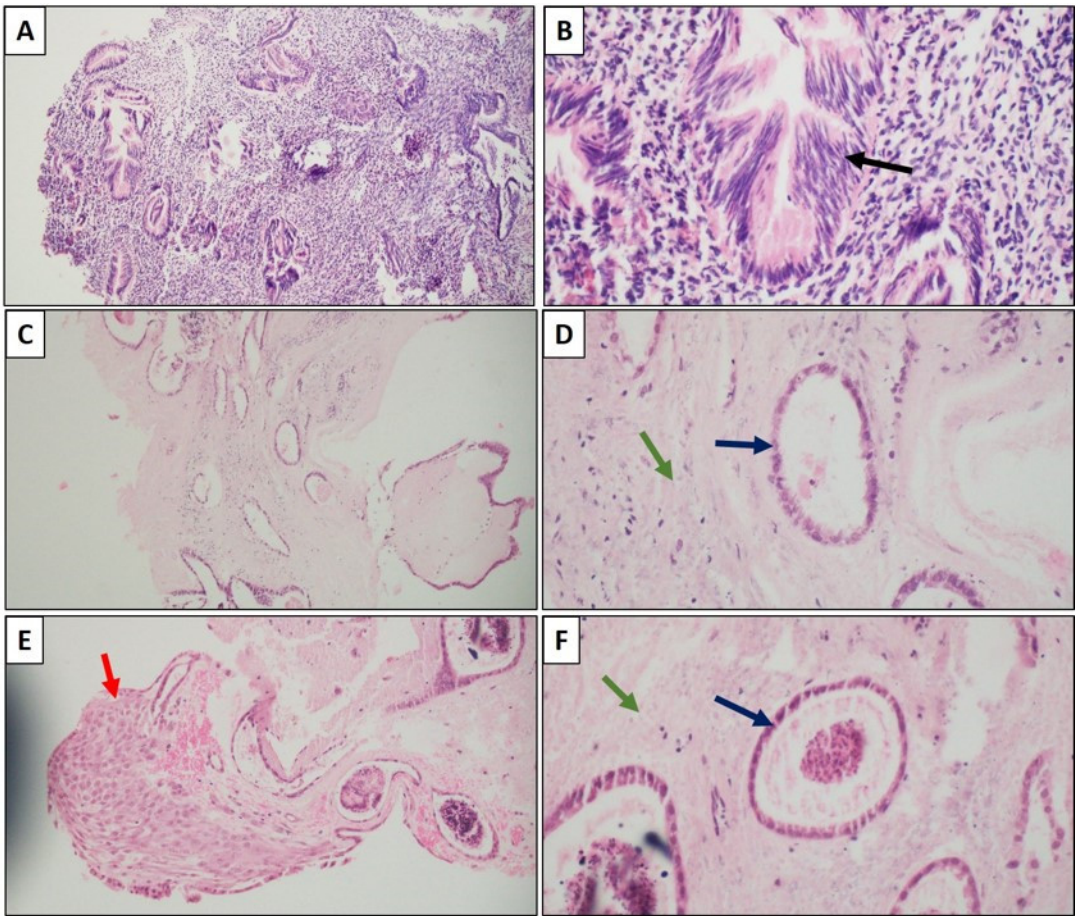


**Supplementary Figure S3.** *Case 3 endometrium – H&E staining, 10× (A, C), 20× (E) and 40× magnifications (B, D, F)*

*A & B: 'Fresh' sample, black arrow shows mechanical injury of endometrial glands (artificial injury)*

*C & D: Control cultured sample, green arrow shows stromal necrosis around endometrial glands (blue arrow)*

*E & F: Hormone treated and cultured sample, red arrow shows squamous cell metaplasia, stromal necrosis (green arrow) can be seen around endometrial glands (blue arrow)*

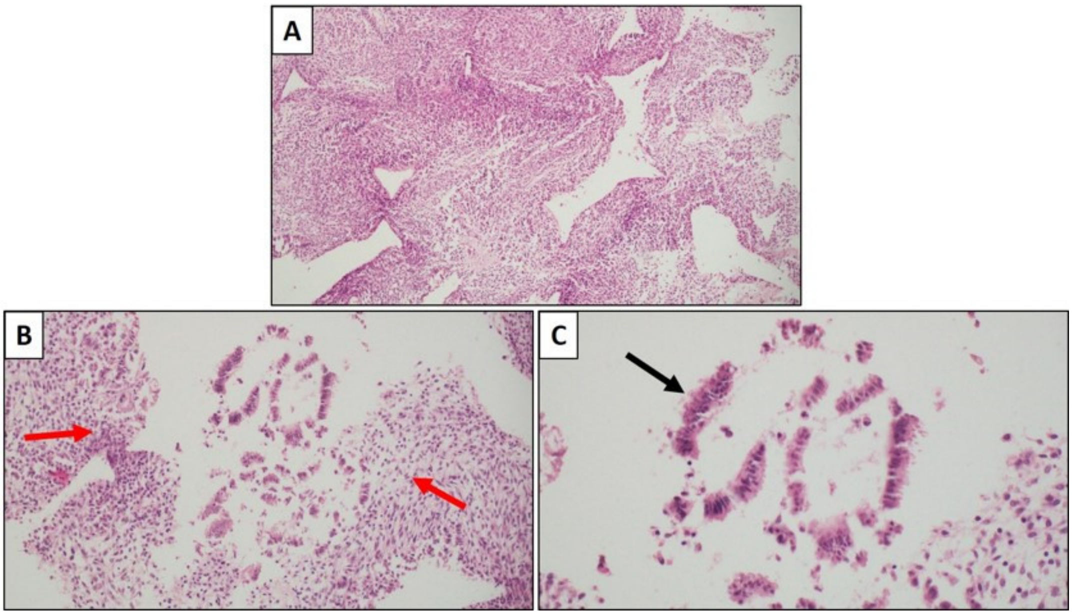


**Supplementary Figure S4.** *Case 4 endometrium – H&E staining, 10× (A), 20× (B) and 40× (C) magnifications of 'Fresh' sample*

*Black arrow shows endometrial gland cells separated from the stroma (red arrows)*

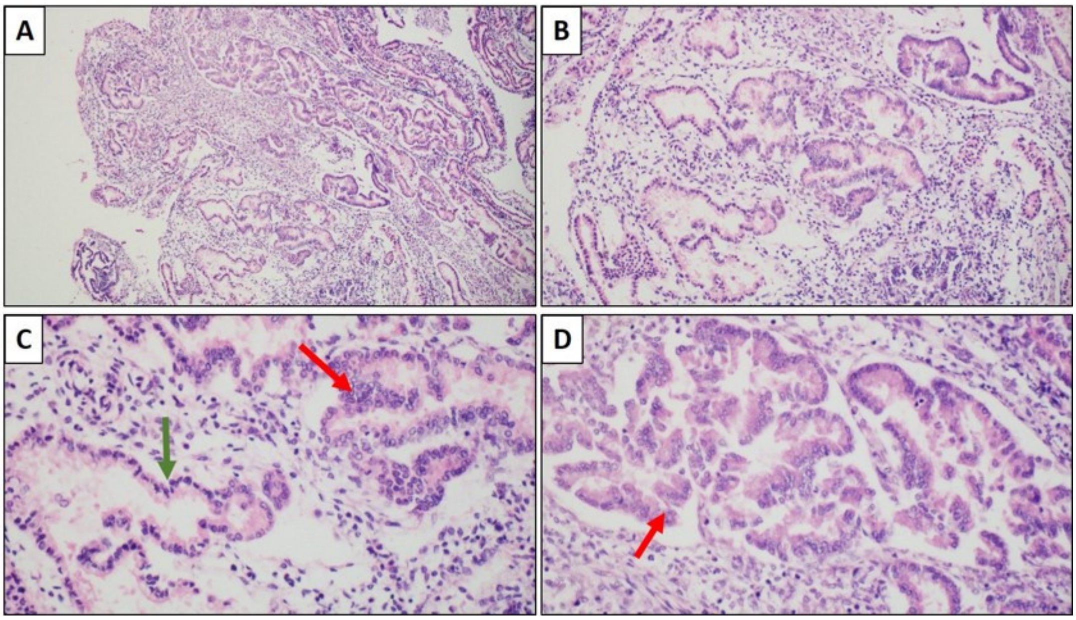


**Supplementary Figure S5.** *Case 5 endometrium – H&E staining, 10× (A), 20× (B) and 40× (C, D) magnifications of 'Fresh' sample*

*Proliferative (green arrow) and secretory phase (red arrow) endometrial glands can be seen alongside each other*

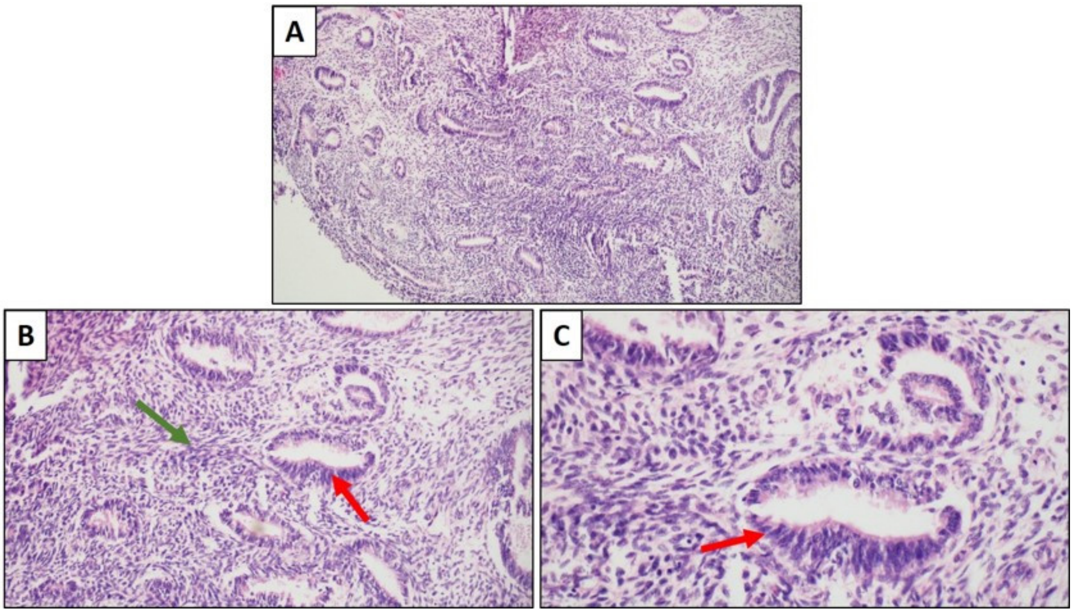


**Supplementary Figure S6.** *Case 6 endometrium – H&E staining, 10× (A), 20× (B) and 40× (C) magnifications of 'Fresh' sample*
*Secretory phase endometrial glands (red arrow) are embedded to the proliferative phase stroma (green arrow)*

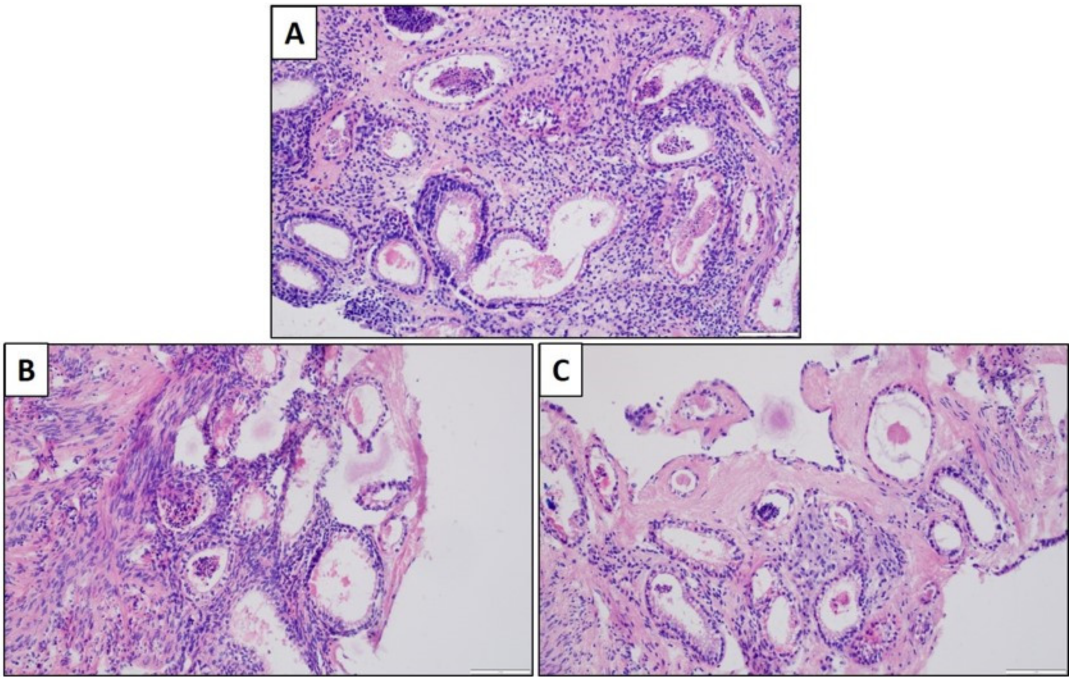


**Supplementary Figure S7.** *Case 7 endometrium – H&E staining, 20× magnifications*
*A: 'Fresh' sample, B: Control cultured sample, C: Hormone treated and cultured sample*

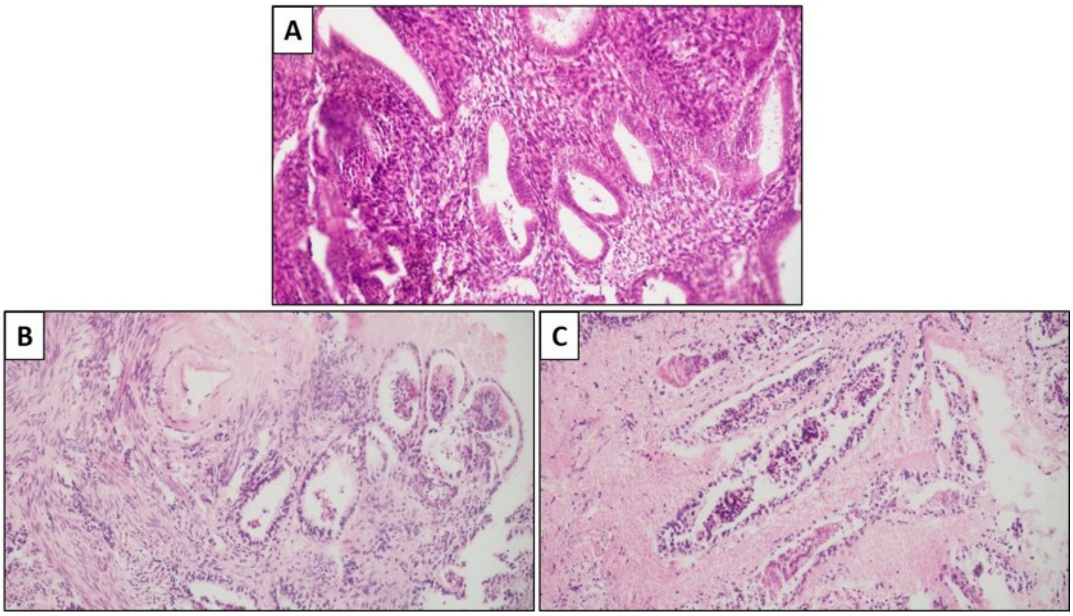

**Supplementary Figure S8.** *Case 8 endometrium – H&E staining, 20× magnifications*
*A: 'Fresh' sample, B: Control cultured sample, C: Hormone treated and cultured sample*
